# Genomic variability correlates with biofilm phenotypes in multidrug resistant clinical isolates of *Pseudomonas aeruginosa*

**DOI:** 10.1038/s41598-023-35056-0

**Published:** 2023-05-15

**Authors:** Ovinu Kibria Islam, Israt Islam, Otun Saha, Md. Mizanur Rahaman, Munawar Sultana, Dirk P. Bockmühl, M. Anwar Hossain

**Affiliations:** 1 Department of Microbiology, University of Dhaka, Dhaka, Bangladesh; 2 Department of Microbiology, Noakhali University of Science & Technology, Noakhali, Bangladesh; 3 Faculty of Life Science, Rhine-Waal University of Applied Science, Kleve, Germany; 4 Present Address: Department of Microbiology, Jashore University of Science & Technology, Jashore, Bangladesh

**Keywords:** Microbiology, Antimicrobials, Biofilms, Clinical microbiology, Microbial communities, Environmental microbiology, Microbial genetics

## Abstract

The multifactorial nature of *Pseudomonas aeruginosa* biofilm development and genomic variabilities implicates its resistance to conventional antimicrobials and virulence. Therefore, genetic determinants need to be extensively studied to block the early steps of biofilm or already formed biofilms. In this study, a total of 20 multidrug resistant (MDR) clinical *P. aeruginosa* isolates were evaluated for their biofilm forming abilities and related genes. Of the isolates tested, all of them showed surface attachment tendencies in nutrient limiting conditions, and classified as strong (SBF = 45%), moderate (MBF = 30%) and weak (WBF = 25%) biofilm formers. Complete genome sequencing of representative strong (DMC-27b), moderate (DMC-20c) and weak biofilm former (DMC-30b) isolates was performed. Analysis of biofilm related genes in the sequenced genomes revealed that, 80 of the 88 biofilm related genes possess 98–100% sequence identity to the reference PAO1 strain. Complete and partial sequence data of LecB proteins from tested isolates indicate that isolates containing PA14-like LecB sequences produced strong biofilms. All of the 7 *pel* operon protein coding genes in weak biofilm former isolate 30b showed significant nucleotide sequence variation with other tested isolates, and their corresponding proteins are 99% identical with the *pel* operon proteins of PA7. Bioinformatics analyses identified divergent sequence and structural features that separate PA7 like *pel* operon proteins from reference PAO1-like *pel* operon. Congo red and pellicle forming assays revealed that the sequence and structure variations may have interfered with the Pel production pathway and resulted in impaired Pel production in isolate 30b that has a PA7 like *pel* operon. Expression analysis also showed that both *pel*B and *lec*B genes were about 5 to 6 folds upregulated after 24 h in SBF 27b in comparison with WBF 30b. Our findings indicate significant genomic divergence in biofilm related genes of *P. aeruginosa* strains that affect their biofilm phenotypes.

## Introduction

Biofilms have become an emerging health problem as they are more resistant to antimicrobials and disinfectants than their planktonic equivalents^1^. Among the biofilm producing bacteria, *Pseudomonas aeruginosa* can cause serious health threats, as it is one of the leading causes of nosocomial infections all over the world^2^. *P. aeruginosa* is one of the most frequent causes of ventilator associated pneumonia and catheter related infection^3,4^. Moreover, in recent times, researchers have witnessed an increasing occurrence of multidrug resistant (MDR) and extensively drug resistant (XDR) *P. aeruginosa* strains. Infections caused by this organism are often associated with high morbidity and mortality due to their outstanding capacity of carrying antimicrobial genes^5–7^.

*P. aeruginosa* strains from infected patients can spread to the hospital environment and their biofilms can be potential reservoirs for disease transmission. Biofilm structures of different *P. aeruginosa* strains can show variability in biomass and morphology^8^. In fact, a number of genes and their products are involved in *P. aeruginosa* biofilm exopolysaccharide secretion, cell to cell signaling and biofilm architecture maintenance^9^. These biofilm-related gene products often interact with each other to give stability to the biofilm matrix. For example, the *pel* and *psl* operons are composed of 7 and 12 genes, respectively. Gene products of these genes are responsible for Pel and Psl polysaccharide synthesis, which has a structural and protective role in the biofilm matrix^10,11^. On the other hand, lectin binding protein LecB is located in the outer membrane and it binds with Psl to stabilize the biofilm matrix^12,13^. As biofilm formation is dependent on various genetic and environmental factors, there is no consistent approach for the control of biofilms and control strategies against biofilms formed by pathogenic bacteria should be directed on a case-by-case basis^14,15^. Genomic and phenotypic variability among biofilm producing strains make anti-biofilm therapy more complicated. Till date several approaches also have been used to block the early step of biofilm formation or to destroy the already formed biofilms^16^. The need for multi-targeted or combinatorial therapies is therefore becoming the focal point to control the multifactorial nature of biofilm growth^17^.

In this work, whole genome sequencing of biofilm forming strains and subsequent bioinformatics analyses of biofilm related genes and proteins can be useful to assess the sequence and structural variabilities that may affect the biofilm forming abilities of different *P. aeruginosa* strains. This study focused on analyzing the sequence variation, protein structure, and expression pattern of biofilm associated genes in MDR clinical *P. aeruginosa* clinical isolates found in Bangladesh. The findings of the study can provide more insights into the genomic variability that affect biofilm phenotypes of *P. aeruginosa* and can support to find effective therapeutics against their biofilms.

## Results

### Biofilm Properties of MDR *P. aeruginosa* isolates

**Figure 1:**
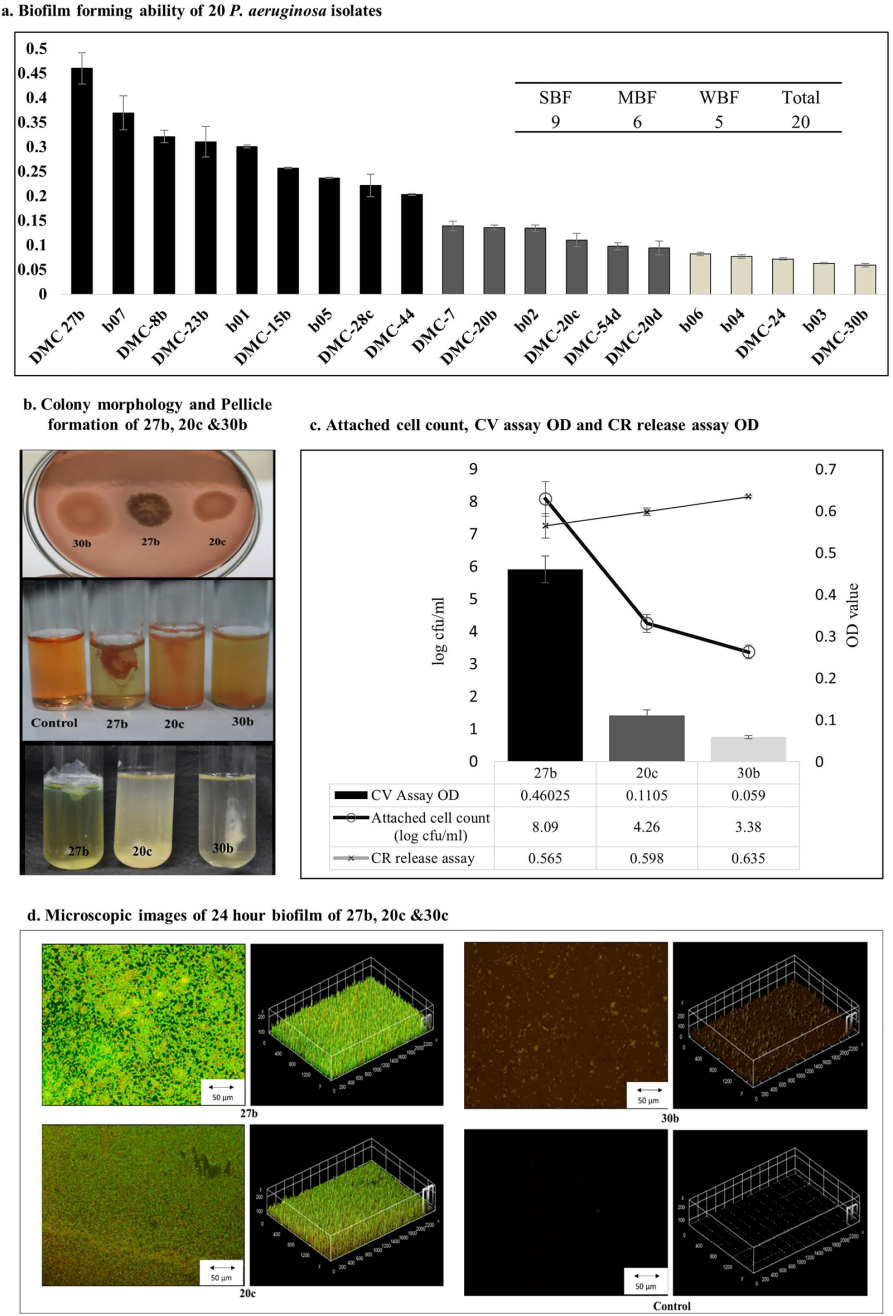
Biofilm phenotypes of different *P. aeruginosa* isolates. (**a**) Bar chart showing the result of Crystal Violet biofilm formation assay of 20 *P. aeruginosa* isolates. The assay was triplicated and the error bar showing the standard deviation from the mean OD for each isolate. (**b**) Colony morphology of SBF, MBF and WBF on Congo red (CR) containing LB agar plates (upper part) showing distinct colony morphology and Congo red release assay showing relative amount of exopolysaccharide produced by biofilms (middle part) and 120 h incubation in LB broth showing pellicle formation in air liquid interface by SBF and MBF isolates (lower part). (**c**) Bar chart showing the CV assay, line chart showing the attached viable cell count and CR release assay OD reading. (**d**) 24 h biofilms of strong (DMC-27b), moderate (DMC-20c) and weak (DMC-30b) biofilm former *Pseudomonas aeruginosa*. For control, 5% TSB was incubated for 24 h without bacterial inoculum. Biofilm was stained with FilmTracer live/dead stain and visualized with Olympus BX53 fluorescent microscope.

Among 20 previously identified and characterized clinical MDR *P. aeruginosa* isolates, 9 were found to be strong biofilm formers (SBF), 6 were moderate biofilm formers (MBF) and 5 were weak biofilm formers (WBF) (Fig. 1a).

On the basis of their distinct biofilm properties (Fig. 1b, c, d), isolates DMC-27b (27b), DMC-20c (20c), and DMC-30b (30b) were chosen as strong biofilm former (SBF), moderate biofilm former (MBF), and weak biofilm former (WBF) representatives for further in-depth molecular analysis. Representative 2D and 3D fluorescent microscopy images of biofilms produced by these isolates showed a clear difference in biofilm structure and attachment pattern to the surface (Fig. 1c, d). The log cfu/mm^2^ counts of viable cells attached to the glass surface were 8.09, 4.96, and 3.38 for isolates 27b, 20c, and 30b respectively. After inoculation in standing LB broth at low cell densities (OD600 = 0.0025), these isolates grew exponentially, and at 120 h, the pellicle of 27b acquired extremely rigid properties and could not be dispersed even by extensive vortexing and boiling. Isolate 20c showed a thin and less rigid pellicle than isolate 27b after 120 h, while isolate 30b showed no visible pellicle in the air liquid interface. On Congo Red (CR) containing LB agar plates, colonies of the isolate 27b had a wrinkled or ‘rugose’ morphology, whereas 20c and 30b colonies were smooth. Dark red colonies of 27b, pink and pale pink colonies of 20c and 30b, respectively, indicated the relative amount of Congo red absorption. The CR release assay also confirmed that the amount of unbound CR released in the media was higher for 20c and the highest for 30b.

### Genomic properties of representative SBF, MBF and WBF isolates

**Figure 2:**
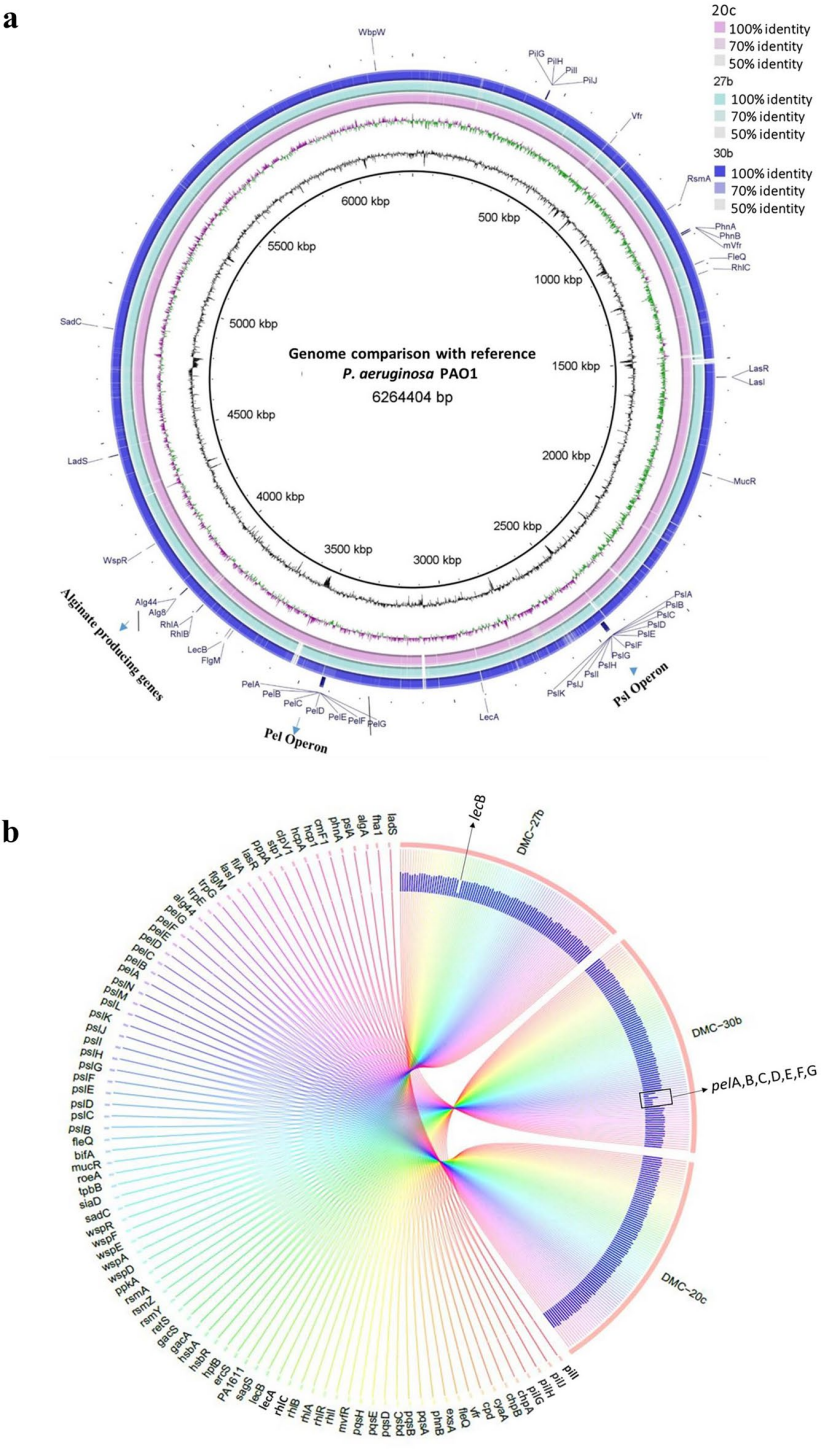
Genomic variation in biofilm related genes of isolate 27b, 20c and 30b.(**a**) Different colors are used to indicate the isolates' mapped genomes. The SBF (27b), MBF (20c), and WBF (30b) strains' percentage of genomic similarities with the reference sequence are indicated by color gradients. Several significant genes related with biofilms have their genomic locations indicated. (**b**) A Circos figure illustrating the percentage of sequence variations in the biofilm-related genes in comparison to the reference PAO1.The lecB and pel operon genes are visibly displaying significant divergence, according to this map. Full data is available on supplementary table 5.

For initial screening, four biofilm related genes (*pel*B, *lec*B*, pil*T*, and rhl*B) were amplified and detected in all of the tested isolates (n = 20) using the primers mentioned in Supplementary Table 2 (data not shown). Complete genome annotation of isolates DMC-27b, DMC-20c, and DMC-30b revealed that, these organisms differ slightly in the GC content and number of coding sequences. According to the PathogenFinder, all three isolates possess a 72–75% chance of being human pathogens. Multilocus sequence typing (MLST) profiling indicated that 27b has a unique sequence type, while 20c and 30b are similar to number 664 and 244 sequence types (ST), respectively. *K*-mer analysis shows that 27b, 20c, and 30b isolates are closely related to the *P. aeruginosa* strains E6130952, PABL012 and W16401, respectively (Supplementary table 3). These isolates' secondary metabolite analyses revealed the presence of the genes for two key biofilm-regulating components (phenazine and homoserine lactone) in all three isolates (Supplementary table 4), However, the whole genome analysis showed that these isolates differ in some sections of their genomes where biofilm-related genes are present (Fig. 2a).

Comparison of 88 proteins and regulatory RNA sequences with reference strain PAO1 revealed that LecB proteins have 13% sequence dissimilarity in SBF isolate 27b and 7 *pel* operon proteins have 5–13% sequence variation in WBF isolate 30b. Other biofilm related proteins and regulatory RNAs showed 98–100% sequence homology with the reference strain. (Fig. 2b, Table 1, Supplementary table 5).

**Table 1:** Comparison of amino acid sequences of LecB protein and Pel operon proteins in sequenced strong, moderate and weak biofilm former isolates (27b, 20c and 30b).

Name of genes/ products	Location	Number of amino acids	Homology		
			27b	20c	30b
fucose-binding lectin PA-IIL, LecB	Unknown	115	99/115 (87%)	115/115 (100%)	115/115 (100%)
PA3064 (pelA)	Unknown	467	466/467 (99%)	466/467 (99%)	884/948 (93%)
PA3063 (pelB)	Unknown	863	853/863 (98%)	1188/1193 (99%)	1042/1193 (87%)
PA3062 (pelC)	Outer Membrane	172	172/172 (100%)	172/172 (100%)	165/172 (95%)
PA3061 (pelD)	Cell Membrane	455	450/455 (98%)	449/455 (98%)	410/455 (90%)
PA3060 (pelE)	Cell Membrane	329	320/329 (97%)	327/329 (99%)	298/329 (90%)
PA3059 (pelF)	Cytoplasmic	507	505/507 (99%)	506/507 (99%)	459/507 (90%)
PA3058 (pelG)	Cell Membrane	456	456/456 (100%)	456/456 (100%)	440/456 (96%)

### Variation in *pel* operon proteins

**Figure 3:**
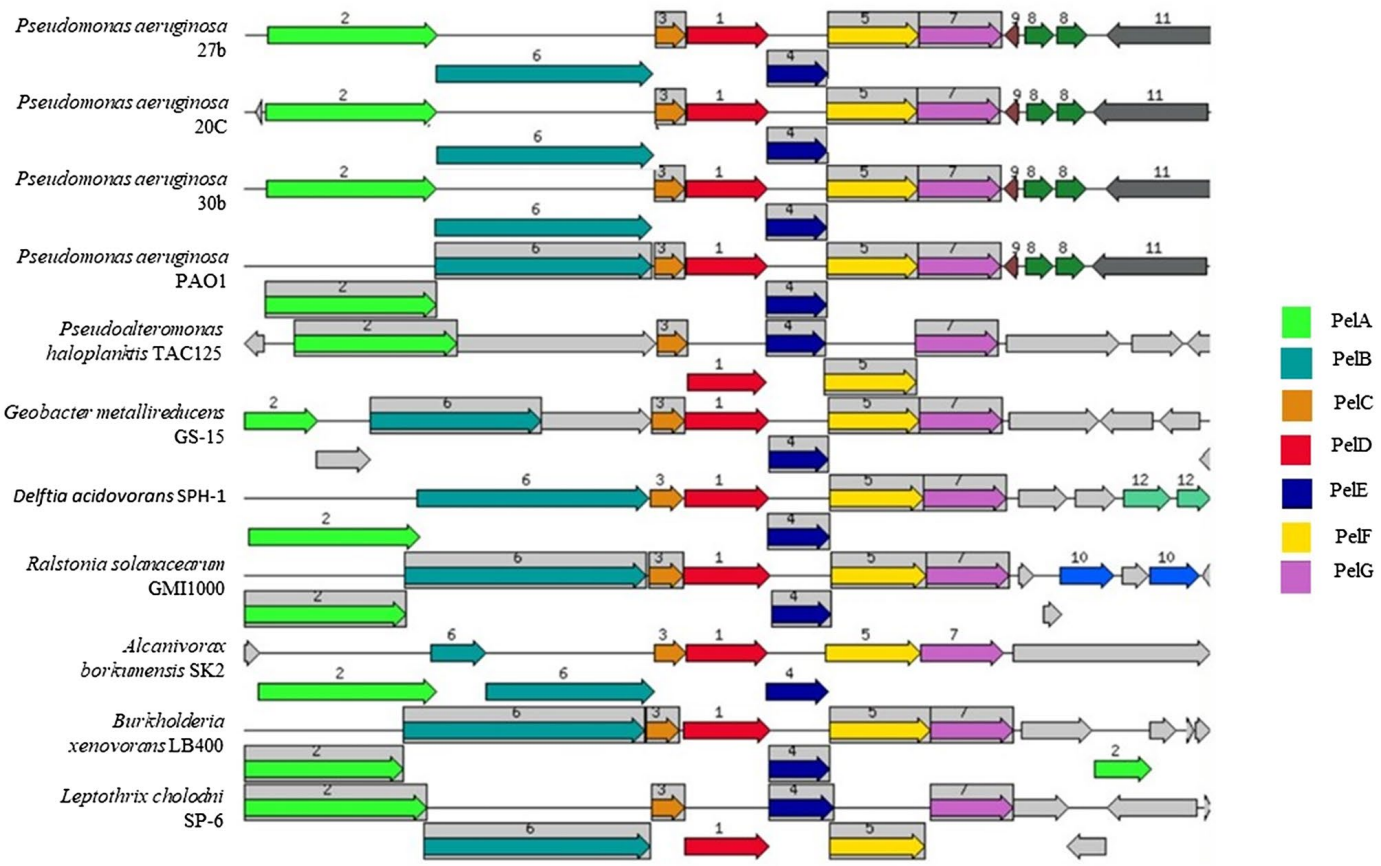
Synteny analysis and gene organization of Pel operon gene clusters. Isolate 27b, 20c, 30b Pel operon gene clusters were compared with *Pseudomonas aeruginosa* PAO1 and other bacterial species containing Pel operon like genes. Genes whose relative position is conserved in at least four other species are functionally coupled and share gray background boxes.

Synteny analysis showed that the genomic organization of the *pel* operon genes of isolates 27b, 20c, and 30b have similarities with *P. aeruginosa* PAO1 in genomic size and orientation despite their sequence variation (Fig. 3). The partial PelB protein (649–692) sequence from 10 clinical isolates, including 27b, 20c, and 30b, reveals that the 30b PelB sequence contains three aa variations (A651T, L658T, and S671N), which are not present in other tested isolates (Supplementary Fig. 3).

**Figure 4:**
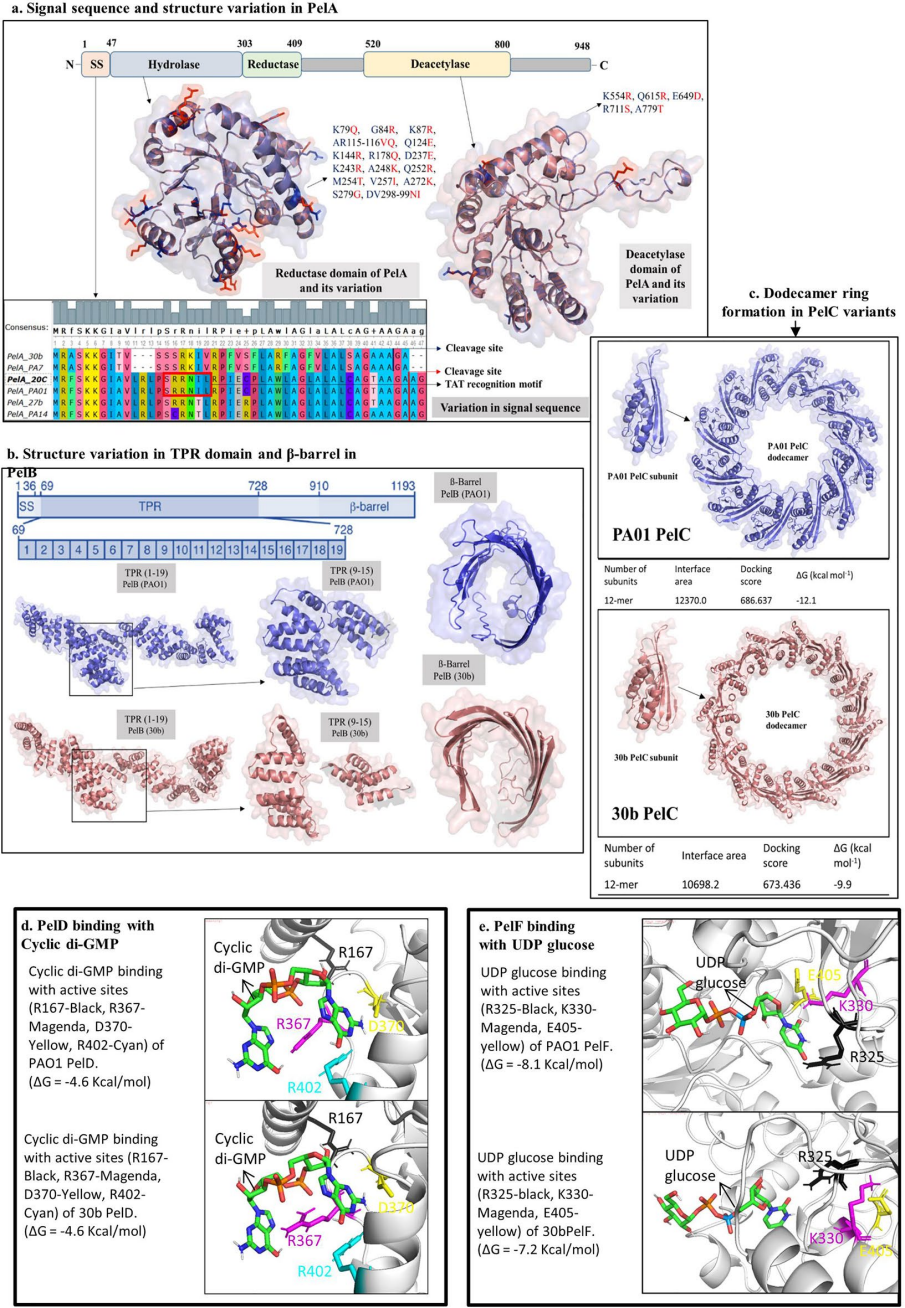
In silico analysis of *pel* operon proteins (**a**) Important enzymatic domains and signal sequence of PelA. Superimposed cartoon and surface representation of the PelA Hydolase domain (47–303) and deacetylase domain (520–800) of 30b and PAO1, showing amino acid variation sites in 30b PelA. Sequence variabilities are also observed in N terminal sequences of PelA in different strains. (**b**) 3D structure prediction of TPR motif region and ß-barrel domain of PAO1 PelB (top) and 30b PelB (bottom). (**c**) Cartoon and surface representation of PAO1 PelC and 30b PelC monomer (19–172) and deodecamer ring structure with homomer docking results. (**d**) C-di-GMP binding with PAO1 PelD and 30b PelD. (**e**) UDP-glucose binding with PAO1 PelF and 30b PelF.

The upstream sequence of the 27b and 20c *pel* operons contains two FleQ binding sites, while the 30b *pel* operon does not have any of them (Supplementary Fig. 4). The TAT recognition motif containing twin arginine residues was found in the N-terminal region of *P. aeruginosa* PAO1, 20c, and 27b, but not in PA7 and 30b (Fig. 4a). We found 18 aa variations between 30b PelA and reference PAO1 PelA hydrolase domain, while 5 aa variations were found in the deacetylase domain (Fig. 4a). The TPR 9–15 region of PelB that interacts with PelA contains 27 aa variations between PAO1 PelB and 30b PelB. 3D modelling also shows significant structure alteration of TPR9-15 motifs (351–588) in 30b PelB (Fig. 4b). The ß-barrel structure of PelB was also found to have conformational changes in 30b PelB according to the *Phyre*^2^ prediction.

Seven aa variants were found between PAO1 PelC and 30b PelC, and four of them occurred between the eighth and eleventh position of PelC. The homo-oligomer prediction tool showed that the 30b PelC dodecamer has a lower interface area and docking score than that of the 27b PelC dodecamer (Fig. 4c). The c-di-GMP binding sites in 30b PelD do not show any conformational changes, and molecular docking reveals that c-di-GMP binds to both PAO1 PelD and 30b PelD with similar PRODIGY scores (ΔG =  − 4.6 and ΔG =  − 4.8 respectively). Nevertheless, other aa variations in both cytoplasmic domains show distinguishable changes in the predicted 3D structure in 30bPelD (Fig. 4d). Molecular docking and corresponding PRODIGY scores suggest that the binding affinity of reference PAO1 PelF with UDP-glucose is higher (ΔG =  − 8.1) than that of 30b PelF (ΔG =  − 7.2) (Fig. 4e). Protein sequence analysis revealed that, PelD, PelE, PelF, and PelG proteins of 30b have 45, 31, 48, and 16 aa variations from similar PAO1 proteins, respectively, while 20c and 27b have between 0–9 aa variations only. The RQ analysis revealed that in a matured biofilm of 24 h, the levels of transcripts of *pel*B of isolate 27b were found to be between 5 and 6 folds more up-regulated than those of 30b (Supplementary table 6).

### LecB protein variation

Isolates 30b and 20c contain a PAO1-like LecB sequence, while isolate 27b contains a PA14-like sequence (Supplementary Fig. 1a). The secondary structure analysis and phylogenetic analysis of related proteins showed that, LecB of 27b have different alpha helix and beta sheet compostion, different solvent accessibility, and is phylogenitically distant from LecB of other two isolates (30b and 20c) (Supplementary Fig. 1c, 2). 3D structure prediction also revealed that, all of the amino acid variations in 27b LecB is located on the outer surface of the protein (Supplementary Fig. 1b). Partial sequencing of the *lec*B gene from 5 SBF and 5 MBF/WBF isolates revealed that all of the SBF isolates possess a PA14 like *lec*B sequence, while MBF/SBF isolates contain a PAO1-like *lec*B sequence (Fig. 1a, Supplementary Fig. 3).

The relative quantification (RQ) of *lecB* transcripts revealed that, in SBF isolate 27b, *lec*B genes were found to be about 5 folds more up-regulated than those of WBF 30b (Supplementary table 6). 27b LecB has two aa variation in the 3-O-alpha-D-Mannopyranosyl-D-mannopyranose binding site (Supplementary Fig. 1d).

## Discussion

Biofilm-forming *P. aeruginosa* strains are one of the major health threats in clinical settings all over the world. Therefore, it is very important to assess the biofilm forming potential and the mechanisms of clinical isolates. Different studies on biofilm formation of clinical *P. aeruginosa* isolates showed variability in the biofilm forming ability around the world^18–20^.

Several genes have been found to play important roles in *P. aeruginosa* biofilm formation. *P. aeruginosa* DNA microarray analysis revealed only 1% of genes that are differentially expressed in the biofilm growth mode compared to free-living^21^. The biofilm formation pathways of *P. aeruginosa* involve 88 genes that encode proteins or regulatory RNAs, according to the Kyoto Encyclopedia of Genes and Genomes (KEGG) pathway database (entry: map02025)^22,23^. Among them, four major pathways (cAMP/Vfr signaling, c-di-GMP dependent polysaccharide synthesis, quorum sensing, and the Gac/Rsm pathway) in *P. aeruginosa* play vital roles in receiving and processing external signals into its regulatory control at the transcriptional, translational, and post-translational levels and thus regulate biofilm formation^24,25^. Freya Harrison’s team showed that, the development of mature, organized biofilm on ex vivo pig lung tissue depends on the Gac regulatory pathway and the generation of the Pel exopolysaccharide^26^ . Another report suggest that, Quorum sensing regulon express most in in vitro transcriptomes of *P.aeruginosa* that grow as biofilms^27^.

We first screened all of the isolates (n = 20) for the presence of four key biofilm genes (*pel*B, *lec*B, *pil*T, and *rhl*B). These genes were initially screened due of their various functions in biofilm development and architectural preservation (e.g., *pel*B in pel synthesis, *lec*B in lectin binding, *pil*T in twitching motility, and *rhl*B in rhamnolipid production). In earlier research, the ability to generate biofilms was found to be compromised or altered due to mutations or deletions of these genes^10,28–30^. All of the tested isolates in our study had these genes, despite having different biofilm morphologies, suggesting that their different biofilm structures were unrelated to the presence or absence of these genes. Thereby, complete genome sequences of isolates exhibiting high, moderate, and weak biofilm formation (27b, 20c, and 30b, respectively) were further investigated to search for any sequence variations in genes that might have contributed in biofilm formation. We compared the genomes of three isolates (27b, 20c, and 30b) with the reference genome of *P. aeruginosa* PAO1 to analyze the 88 protein- and regulatory RNA-coding genes associated with biofilm development. Analysis of antibiotic resistant genes and gene cassettes of 27b from its CGS was previously reported by Jahan et al.^31^ and genomic diversity and molecular epidemiology of 30b was reported by Hoque et al.^32^. Only the LecB and pel operon proteins among those 88 biofilm-related proteins and RNAs displayed notable aa sequence divergence in neucleotide and aa sequences in isolates 27b, 20c, and 30b (Fig. 2 and Supplementary table 5).

The *pel* operon plays a vital role in the synthesis of Pel, which is an aggregative polysaccharide produced by *P. aeruginosa*. This operon encodes seven enzymes (PelA, PelB, PelC, PelD, PelE, PelF, PelG)^33^. Individual BlastN searches in the NCBI nucleotide database of individual *pel* operon genes of isolates 27b, 20c, and 30b revealed that all of the *pel* operon genes of 30b have a certain amount (5–13%) of sequence variation from the majority of other *pel* operon genes in the database, and only 6 strains share 98–100% sequence identity with *Pel* operon genes of 30b (Supplementary Table 6). A BlastP search in the NCBI standard protein database revealed that 30b *pel* operon proteins share sequence homology with *P. aeruginosa* PA7 *pel* operon proteins (data not shown). Based on these findings, we propose two types of *pel* operons: PAO1-like (reference) and PA7-like (variant).

According to reports, strains that overexpress pel polysaccharide have rugose colony shape, thick pellicle at the air–liquid interface, and aid in congo red binding^10,33,34^.

The colony morphology, microscopic observation, pellicle forming and congo red assays (Fig. 1b,c,d) revealed that, WBF 30b is unable to produce pel polysaccharide. On the other hand, genomic sequence analysis of the 30b *pel* operon revealed significant variation in comparison with the reference PAO1 *pel* operon. Potentially, the synthesis of pel might be impaired by these genetic variations.

Recent studies have also described a role for the flagellum regulator FleQ as both a repressor and an activator to control gene expression from the *pel* operon promoter in response to c-di-GMP^35^. The absence of these FleQ binding sites may play a vital role in the expression of *pel* operon proteins. In this study, we found that FleQ binding sites are absent in the upstream of the 30b (PA7 like) *pel* operon. Although, the regulator FleQ binding sites are absent in the upstream of the 30b *pel* operon, we found the presence of *pel*B transcripts with a common *pel*B primer (Supplementary Table 2), which indicates *pel* operon proteins might have been expressed in 30b.

It was previously reported that PelA has a tat-dependent signal sequence, suggesting the protein is localized to the periplasm^36^*.* Our findings indicate that PelA of 30b cannot be transported across the cytoplasmic membrane via the Tat secretion machinery as it does not have the twin arginine residue in its tat recognition motif. It was also predicted that, at least four, and possibly five, distinct domains, three of which have structural similarities to proteins with hydrolase, reductase, and deacetylase activity are present in PelA^36^. aa variations in those protein domains of 30b may affect the function of PelA.PelA and PelB are known to directly interact with one another. The TPR-containing domain of PelB localizes PelA to the Pel polysaccharide secretion apparatus within the periplasm^37^*.* When pel is deacetylated by pelA, it becomes positively charged. As a result, Pel is drawn toward the electronegatively charged PelC, which guides Pel toward the exit channel formed by PelB. In this proposed model, PelC functions as an electronegative funnel by forming a dodecamer ring around the ß-barrel domain of PelB(^38^. Our 3D structure analysis predicted conformational changes in the ß-barrel of the TPR domain in 30b P*elB* (PA7 like PelB)*.* The development of the PelC dodecamer ring appears to be affected by aa changes in 30b PelC (PA7-like PelC), according to in silico modeling.

PelD, PelE, PelF, and PelG are responsible for pel polymerization and transport across the cytoplasmic membrane^39,40^. It was also previously reported that c-di-GMP functions post-translationally in Pel synthesis by modulating the activity of PelD. R161, R367, D370, and R402 are the 4 aa’s that interact with c-di-GMP, which are present in all of our sequenced isolates (27b, 20c, and 30b). On the other hand, PelF uses UDP-glucose as a donor substrate toward the biosynthesis of the Pel exopolysaccharide. PelF's E405, R325 and K330 are proposed to be its UDP glucose binding sites^39^.

According to the results of our in silico investigation, 30b PelF (PA7-like PelF) has a lower binding affinity for UDP glucose than PAO1-like PelF.

**Figure 5:**
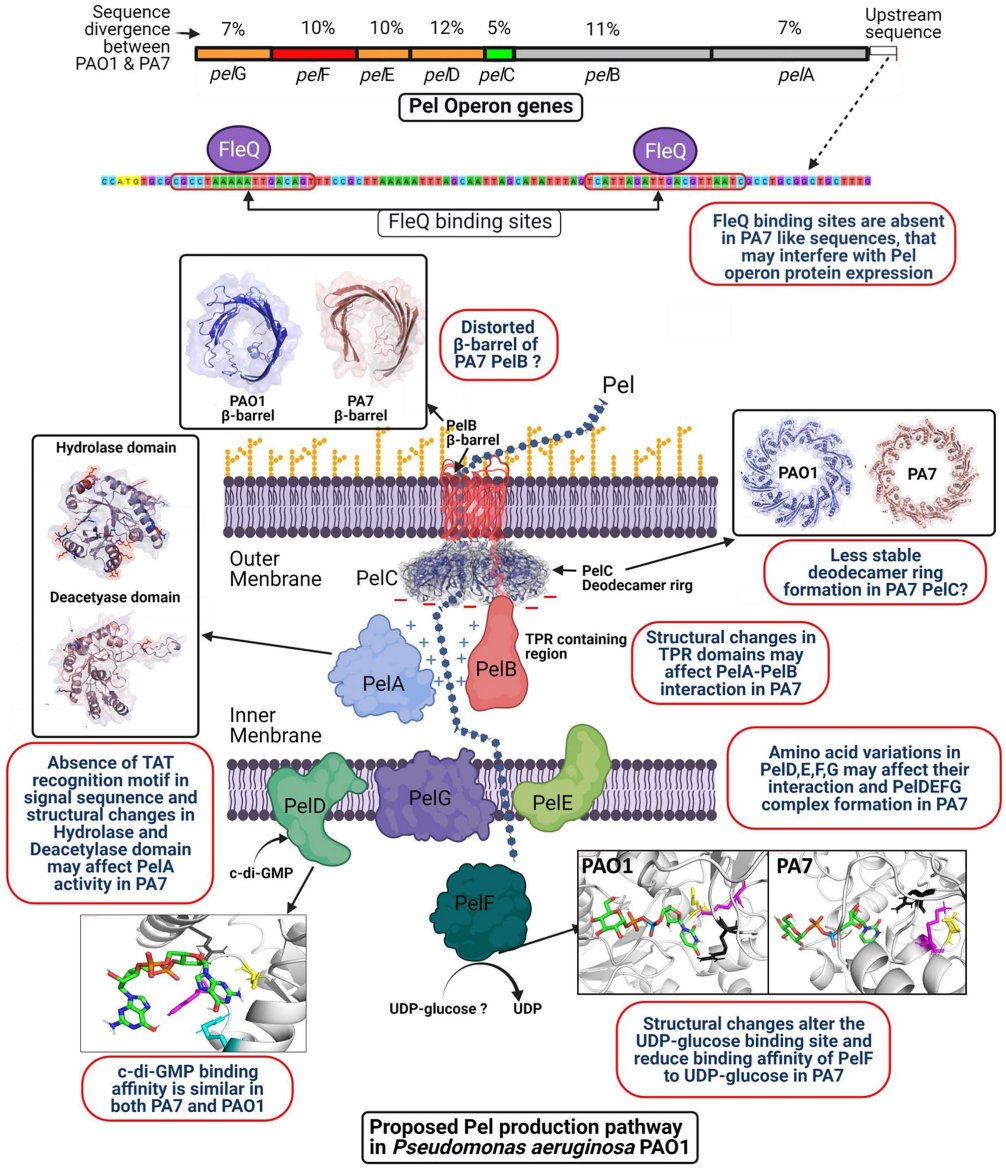
Schematic diagram showing proposed Pel production pathway in *Pseudomonas aeruginosa* PAO1. This figure also illustrates the sequence variation between PAO1 and PA7 strains and probable effects of the sequence divergence on Pel production process.

Moreover, aa divergence in the PA7 gene, such as PelD, PelE, PelF, and PelG, may influence their interaction. In Fig. 5, we summarized the possible reasons that can adversely affect Pel production machinery in PA7 like *pel* operon possessing strains. Isolate 27b and 20c have PAO1-like *pel* operon sequences, and both of them were able to produce Pel polysaccharide. Our findings based on in silico analysis suggest that PA7 like *pel* operon containing strains are unable to produce Pel polysaccharide as a component of their biofilm matrix.

According to a previous report^41^, LecB from the highly virulent model strain PA14 has a 13% sequence divergence with LecB from the well-characterized PAO1 strain. According to our partial sequence data (Supplementary Fig. 3) and the related microtitre plate biofilm formation investigation (Fig. 1a), isolates with LecB that are similar to PA14 are strong biofilm formers, whereas isolates with LecB that are similar to PAO1 are either moderate or weak biofilm formers. To our knowledge, no study has yet connected this sequence divergence to the biofilm forming ability on abiotic surfaces. One study regarding this revealed that LecB binds with 3-O-alpha-D-Mannopyranosyl-D-mannopyranose of Psl and thus has a profound impact on biofilm architecture and biomass in PAO1^13^. PA14 like LecB differs from PAO1-like LecB in Psl binding site at positions 24 (PA14 LecB Ser; PAO1 LecB Ala) and 98 (PA14 LecB Ser; PAO1 LecB Gly**)** (Supplementary Fig. 1d)**.** It can be noted that PA14 strain itself cannot produce Psl, but other strains that have PA14 like LecB can produce Psl. Variation in the Psl binding site in Psl-producing strains may have an impact on biofilm architecture. Further investigation is therefore necessary to validate this hypothesis.

The main limitation of our study is that we mainly focused on genomic variance and in silico modeling of important biofilm related proteins. In vitro analysis of the proteins we discussed here can give us more conclusive information about the correlation of biofilm forming ability and sequence variation of LecB and *pel* operon proteins.

Our study suggests that MDR clinical *P*. *aeruginosa* isolates from Bangladesh differ in their biofilm phenotypes. Variation in aa sequences in the lectin binding protein LecB and the Pel polysaccharide producing operon proteins in those isolates was found to be related to their biofilm phenotypes. PA14-like LecB protein sequences correlate with increased biofilm formation, while PA7-like *pel* operon sequences correlate with decreased or impaired Pel polysaccharide production. Strain-family classification of *P*. *aeruginosa* is therefore important to understand the multifactorial nature of biofilm formation and to introduce effective therapeutics against them.

## Methods

### Clinical isolates

A total of 20 previously identified and characterized clinical isolates were retrieved from glycerol stocks preserved at -20 °C in the Microbial Genetics and Bioinformatics Laboratory, Department of Microbiology, University of Dhaka^42^. These isolates were selected on the basis of their resistance to antibiotics, and all of the selected isolates were previously found as resistant to at least 3 antibiotic groups. The isolates were isolated from 4 different sources of wound swab, urine, pus, blood and tracheal aspirate samples that were previously collected from Dhaka Medical College Hospital during 2 different sessions—October, 2015 and March, 2016; and from the Bangladesh Institute of Health Science (BIHS) during February–March, 2018 (Supplementary Table 1).

### Biofilm assays and microscopy

Crystal Violet biofilm formation assay (CV assay) was performed using the methods previously described by George O’Toole^43^. 100 µl of diluted culture for each isolate were inoculated into Thermo Scientific™ 96-Well Microtiter Microplates (quadruplicates) for each isolate. The biofilms were then stained with 125 μL of a 0.1% of crystal violet in water. UV absorbance was measured at 595 nm in micro plate reader (Multiskan, Thermo Labsystems) using 30% acetic acid in water as the blank. Biofilm formation ability of isolates was determined by the standard formula (OD ≤ OD_cut_ = Non-biofilm-former, OD_cut_ < OD ≤ 2 × OD_cut_ = Weak biofilm-former, 2 × OD_cut_ < OD ≤ 4 × OD_cut_ = Moderate biofilm-former, OD > 4 × OD_cut_ = Strong biofilm-former and OD_cut_ = OD_avg_[average of OD’s] of negative control + 3 × standard deviation of ODs of negative control. Here OD means optical density of the samples in CV assay)^44,45^.

For Pellicle forming assay, standing cultures containing 3-ml of LB broth (Thermo Fisher Scientific, USA)were grown at room temperature in an 18 × 150 mm Durex™ borosilicate glass tube. Pellicles were monitored by visual inspection between 24 to 120 h. Complete coverage at the air–liquid interface of an opaque layer of cells was considered to be indicative of pellicle formation^34^. To observe colony morphology, overnight cultures were diluted at an OD580 of 0.08 in LB (8.107 CFU.ml^−1^) and 5 μl were spotted onto a LB plate containing 40 μg.ml^−1^ of Congo Red(CR) and 20 μg.ml^−1^ Coomassie brilliant blue as described by Friedman and Kolter^34^. Plates were incubated at 37◦C overnight before visual inspection of the colony morphology. To quantify the CR binding on bacterial cells, a CR release assay was performed as previously described by Lee and his teammates^46^.

A method previously described by Haibo Mu et al. was adapted and slightly modified to generate biofilm on the glass coverslips^47^*.* Biofilms were formed on pre-sterilized microscopic glass coverslips (2mm^2^) (Labtex, Bangladesh) that were placed into a 12 well culture plate (Thermo Fisher Scientific, USA) for a 24 h, incubated at room temperature. Diluted (1/20) tryptic soy broth (TSB, Thermo Fisher Scientific, USA) was used as a nutrient source, and no supplement was used to promote the growth of biofilms. The commercially available LIVE/DEAD® BacLightTM Bacterial Viability Kit (Invitrogen) was used to stain biofilms in accordance with the manufacturer's instructions (Probes 2004) and the procedures outlined earlier by Delben et al.^48^. Biofilm images were taken using an Olympus BX53 upright fluorescence microscope (Olympus, Japan) and a DP73 digital camera with the objective UPLFLN 40X lens (Olympus, Japan). The experiment was conducted in duplicate on two independent occasions.

To remove the cells attached to the surface, sonication was carried out for 1 min at 40 kHz frequency (Citizen Scale Ultrasonic Cleaner YJ5120-1)^49^. The detached cell suspensions were serially diluted in 0.85% NaCl solution, and viable cell counts were determined using the method of Miles et al.^50^by spotting appropriate dilutions (10 µl) onto TSA and incubating them at 37 °C before enumeration.

### Screening and expression analysis of biofilm genes

Primers specifically designed for the *pel*B, *lec*B, *pil*T, and *rhl*B genes were used for the initial screening for genes relevant to biofilms (Supplementary table 2). Amplification was performed in a thermocycler (Applied Biosystems, Foster City, USA) in a total volume of 20 μl containing 10 μl of master mix 2X (Go Taq Colorless Master Mix), 1 μl (1 pmol/1 μl) of each forward and reverse primer, and 2 μl of DNA using the following conditions: initial denaturation of 94° C for 5 min, followed by 30 cycles of denaturation 94° C for 1.0 min, primer annealing at 57° C for 1.0 min, extension at 72° C for 2.0 min and a final delay at 72° C for 5 min. The PCR products were resolved on a 1.5% agarose gel, stained with ethidium bromide (5 μg/ml) and bands were visualized by Gel documentation (protein sample, USA)^51^.

For quantification of *pel*B and *lec*B transcripts, total RNA was extracted from the culture of *P. aeruginosa* isolates using the PureLink™ RNA Mini Kit (Thermo Fisher Scientific, USA) in accordance with the manufacturer*’*s instructions. The quantity of extracted RNA was determined by A260 measurements. The purity (A260/A280) of RNA was > 1.8 when measured with the NanoDrop 2000 spectrophotometer (Thermo Fisher Scientific, USA). To prepare cDNA, experimental RNA was combined with the random and oligo(dT)_15_ primer. The primer/template mixture was thermally denatured for 5 min at 70 °C using a heat block (Veriti 96 well Thermal cycler, Applied Biosystems, USA) and chilled on ice. Reverse transcription reaction mix (30 μl of total volume) containing 9.6 µl nuclease-free water, 8.0 µl 5X reaction buffer, 2.0 µl ImProm-II™ reverse transcriptase, 6.4 µl magnesium chloride (8 mM), 2.0 µl dNTP mix (final concentration 1.0 mM for each dNTP) and 2.0 µl ribonuclease inhibitor was prepared. The PCR conditions used to prepare cDNA was 25 °C for 5 min, 42 °C for 60 min and 85 °C for 10 min^52^.

The quantitative PCR (qPCR) was performed using an SYBR Green qPCR kit (Roche Diagnostics, USA) and the Applied Biosystems 7300/7500 Real Time PCR System (7300/7500 system) was used to measure the relative transcript levels of the *pelB* and *lecB* genes in strong and weak biofilm former isolates. A total of 25 μl reaction volume was used, including 2.5 μl cDNA, 12.5 μl of (2x) GoTaq® qPCR Master Mix, 0.25 μl each of forward and reverse primers, and nuclease-free water to make up the volume. The templates were amplified with an initial denaturation of 95 °C for 2 min, followed by 40 cycles of 95 °C for 15 s, 55 °C for 60 s, and 72 °C for 30 s. The qPCR was performed in triplicates to minimize any errors caused by handling. Relative gene expression (fold change) was calculated using the formula 2^−ΔΔCT^ using *gyr*A gene as control^53^.

### Partial sequencing of biofilm genes

For partial sequencing of *pelB* and *lecB* genes, PCR positive samples were purified using The Wizard® SV Gel and PCR Clean-Up System (Promega, USA) according to the manufacturer’s instructions. The concentration of amplicons was measured using a NanoDropTM spectrophotometer (Thermo Fisher Scientific Inc., Wilmington, DE, USA). After purification of the PCR products, the sequencing reaction was performed for both forward and reverse primers using the BigDye® Terminator v3.1 Cycle Sequencing Kit (Applied Biosystem, ThemoFisher Scientific, Inc., USA). The sequences (tracer files) were viewed using the sequence viewer software Chromas Pro. Both forward and reverse sequences were assembled into a single contig using SeqMan version 7.0.0 (Lasergene, DNASTAR, USA)^54^.

### Whole genome sequencing and analysis

For WGS, the genomic DNA of the isolates was extracted using the QIAamp DNA Mini Kit (Qiagen, Hilden, Germany). Genomic quality and quantity were assured and WGS was done under Ion Torrent platform using 400 bp read chemistry. The Ion Torrent platform generated FASTQ reads quality was assessed by the FastQC tool (Babraham Bioinformatics-FastQC) followed by trimming of low-quality reads and reads less than 200 bp using the Trimmomatic 0.36 version, where quality cut off value was Phred-20^55^. *De-novo* assembly of the reads was performed using SPAdes (version 3.5.0) genome assembler^56^. Assembled contigs were mapped and reordered according to a reference sequence of the *P. aeruginosa* PAO1 genome from NCBI (accession number: NC_002516.2) by the progressive Mauve algorithm in Mauve software^57^. Complete genome sequence (CGS) data of DMC-27b (Accession- NZ_SMRY00000000.2) and DMC-20c (Accession-NZ_JAGRPY000000000.1) and DMC-30b (Accession- NZ_JAMQYG000000000.1) isolates were submitted to the NCBI database. The assembled draft genome of the isolates DMC-27b, DMC-20c, and DMC-30b was annotated by RAST version 2.0^58^. The SEED viewer^59^ was used for the exploration and comparative analysis of annotated genes. Assembled contigs of complete genome sequences were analyzed by KmerFinder 3.0 tool to identify the bacterium at species level^60^. To compare the sequenced genomes of DMC-27b, DMC-20c, and DMC-30b with the reference genome of *P. aeruginosa* PAO1, Blast Ring Image Generator (BRIG) software (Version 0.95)^61^ was used. The circular image of the sequenced genomes was constructed, and these three genomes were marked with different colors. Some important biofilm related genes and operons were also marked, and the similarity of the genomic regions was indicated by a color gradient.

### In silico analysis of biofilm related proteins

From the *Pseudomonas* genome database (www.pseudomonas.com)^62^, the nucleic acid and protein sequences of the biofilm related genes/proteins of *P. aeruginosa* PAO1 (reference genome) were retrieved. The nucleic acid and protein sequences were compared with the annotated genomes in the SEED viewer to determine the homology and variation in the genes/proteins among the studied isolates. This server was also used to perform Synteny analysis of the desired genes and proteins. The pathogenic profiles of the sequenced genomes were determined by PathogenFinder^63^. Secondary metabolite gene clusters were identified by anti-SMASH version 4.0.2 software^64^. The KEGG MG Mapper tool^22,23^ was used for biofilm pathway reconstruction.

The secondary structures of LecB and *pel* operon proteins of sequenced isolates were predicted by the PredictProtein^65^ web tool. The 3D structures of the LecB and *pel* operon proteins of the sequenced genomes were constructed using Phyre 2^66^, and the variation in the protein structures among those isolates was visualized with PyMOL (TM) 2.4.0 software^67^. Protein–ligand docking was performed using the HADDOCK webtool version 2.2^68^. Protein–ligand binding affinities were measured by the PRODIGY-LIGAND web server^69^. For PelC dodecamer structure prediction, the GalaxyHomomer webserver was used. This server predicts the homo oligomer structure of protein from a monomer sequence or structure^70^. The aligned protein sequences were used for the construction of a protein tree using the maximum-likelihood method in the Molecular Evolutionary Genetics Analysis (MEGA X) software^71^. Interactive Tree Of Life (iTOL) v5^72^ was used to adjust the branch and label color of the phylogenic tree. Conceptual figure of Pel polysaccharide formation was created using Biorender Webtool (biorender.com).

## Supplementary Information

Supplementary Information.

## Data Availability

The WGS data of *P. aeruginosa* DMC-27b, DMC-20c and DMC-30b are deposited at DDBJ/ENA/GenBank under accession number NZ_SMRY00000000 (Biosample SAMN10765885), NZ_JAGRPY000000000 (Biosample SAMN18739953)and JAMQYG000000000 (BioSample SAMN28906490) respectively, and the assembly reports of the genome are also available at GenBank (https://ncbi.nlm.nih.gov/nuccore/NZ_SMRY00000000, , https://ncbi.nlm.nih.gov/nuccore/NZ_JAGRPY000000000, https://ncbi.nlm.nih.gov/nuccore/JAMQYG000000000). The versions described in this paper is version SMRY00000000.2, JAGRPY000000000.1 and JAMQYG000000000.1. The Ion Torrent FASTQ reads are available for these isolates (DMC-27b, DMC-20c and DMC-30b) in the NCBI under BioProject accession number SAMN10765885, PRJNA224116 and PRJNA846956 respectively.
