# Supplementary material for: Genomic variability correlates with biofilm phenotypes in multidrug resistant clinical isolates of Pseudomonas aeruginosa
Source: Sci Rep. 2023 May 15;13:7867. doi: 10.1038/s41598-023-35056-0 (PMC10185581; doi:10.1038/s41598-023-35056-0)
Supplement: Supplementary file 1 — Supplementary Information. [file 41598_2023_35056_MOESM1_ESM.pdf]

Hospital	Isolate ID	Source
**DMCH**	27b	Urine
**DMCH**	8b	Wound Swab
**DMCH**	23b	Tracheal Aspirate
**DMCH**	15b	Pus
**DMCH**	DMC-54d	Wound Swab
**DMCH**	DMC-20b	Wound Swab
**DMCH**	DMC-20c	Wound Swab
**DMCH**	DMC-7	Pus
**DMCH**	DMC-28c	Wound Swab
**DMCH**	DMC-30b	Pus
**DMCH**	DMC-20d	Wound Swab
**DMCH**	DMC-44	Pus
**DMCH**	DMC-24	Wound Swab
**BIHSH**	b01	Wound Swab
**BIHSH**	b02	Wound Swab
**BIHSH**	b03	Pus
**BIHSH**	b04	Wound Swab
**BIHSH**	b05	Wound Swab
**BIHSH**	b06	Wound Swab
**BIHSH**	b07	Wound Swab

*DMCH- Dhaka Medical College Hospital

*BIHSH- Bangladesh Institute of Health Science Hospital

Supplementary Table 1- Source of samples

Primer	Sequence (5'->3')	Tm	Length (bp)	Amplicon size	Reference
*PilT*_F_	TCCACGAGTCGAAGAAGTGC	60	20	145	This study
*PilT*_R	AGGCGAATGGTTTCCAGGTC	60	20		
*lecB*_F	CAAGGAGTGTTCACCCTTCC	57	20	306	This study
*lecB*_R	GTCGTTGTAGTCGTTGTCGG	57	20		
*PelB*_F	ACGCCTGCTCTGGTTCTAC	58	19	186	This study
*PelB*_R	TTGGGATTGGACTTGAGGTA	58	20		
*RhlB*_F	CGCTGCTTGTCGTAATCCAC	59	20	96	This study
*RhlB*_R	GGCCATCCAGATCCACAAGG	60	20		

Supplementary Table 2: Primers used in this study for the detection of biofilm associated genes in *Pseudomonas aeruginosa*

	27b (SBF)	20C (MBF)	30b(WBF)
**Size**	6,952,319	7,018,827	7,211,991
**GC Content**	65.8	65.9	65.7
**Number of Subsystems**	432	429	432
**Number of Coding Sequences**	7552	7112	7565
**Number of RNAs**	65	66	66
**Pathogen Probability**	0.727	0.751	0.749
**MLST ST**	New	664	244
**Predicted strain**	E6130952	PABL012	W16401

Supplementary Table 3- Overview of the genomic profile of 3 *Pseudomonas aeruginosa* isolates with pathogen probality, MLST profiling and Kmer analysis.

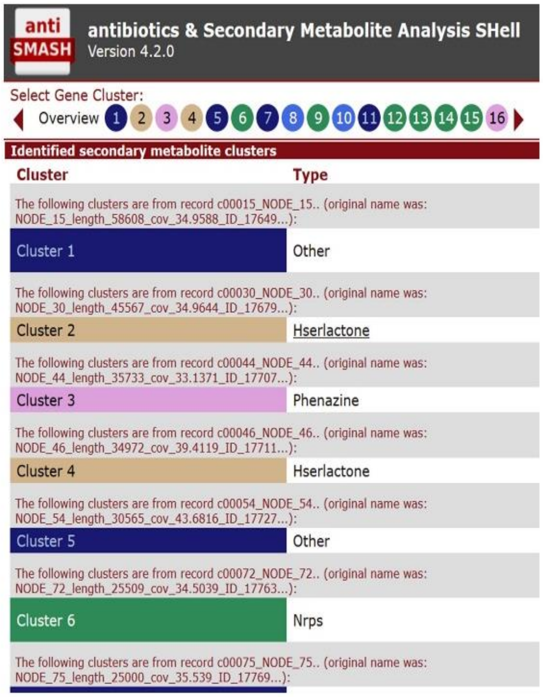

Metabolites	27b	20c	30b
Homoserine lactone	+	+	+
Phenazine	+	+	+
Bacteriocin	+	+	+
Non ribosomal peptides	+	+	+
Arylpoleyene	-	-	+
Mangotoxin	-	-	71%
Pyocyanin	-	-	42%
Pyochelin	40%	40%	-

15

Supplementary Table 4: Secondary metabolite profile of the 3 isolates.

Name of the Genes/ Gene Products	Identity DMC-27b	Identity DMC-30b	Identity DMC-20c
pilI; twitching motility protein PilI	100	100	100
pilJ; twitching motility protein PilJ	99	100	99
pilH; twitching motility protein PilH	100	100	100
pilG; pilus biosynthesis/twitching motility protein PilG	100	100	100
chpA; chemotactic signal transduction system protein	98	99	99
chpC; chemotaxis protein	98	98	98
cyaB; protein CyaB	99	100	100
cpdA; cAMP phosphodiesterase	98	99	99
vfr; cAMP-regulatory protein	100	100	100
fleQ; transcriptional regulator FleQ	100	100	100
exsA; exoenzyme S transcriptional regulator ExsA	100	100	100
phnB; anthranilate synthase component II	99	99	99
pqsA; anthranilate--CoA ligase	99	100	100
pqsB; hypothetical protein	98	100	99
pqsC; hypothetical protein	99	99	100
pqsD; 3-oxoacyl-ACP synthase	99	99	99
pqsE; thioesterase PqsE	100	100	100
pqsH;	100	100	100
mvfR; transcriptional regulator MvfR	99	99	99
rhlI; acyl-homoserine-lactone synthase	99	99	99
rhlR; transcriptional regulator RhlR	100	100	100
rhlA; rhamnosyltransferase subunit A	99	99	98
rhlB; rhamnosyltransferase subunit B	100	100	99
rhlC; rhamnosyltransferase	100	99	99
lecA; PA-I galactophilic lectin	99	100	99
fucose-binding lectin PA-IIL, LecB	87	100	100
sensor histidine kinase SagS	99	100	100
hybrid sensor kinase, PA1611	99	99	99
ercS'; sensor histidine kinase	99	99	99
histidine phosphotransfer protein HptB	99	99	99
biofilm regulator HsbR	99	100	100
anti anti-sigma factor HsbA	100	100	100
gacA; response regulator GacA	100	100	100
gacS; sensor/response regulator hybrid protein	99	100	100
retS; sensor histidine kinase MifS	99	99	99

**Name of the Genes/ Gene Products**	**Identity DMC-27b**	**Identity DMC-30b**	**Identity DMC-20c**
rsmZ; regulatory RNA RsmZ	100	100	100
rsmA; carbon storage regulator	100	100	100
ppkA; serine/threonine protein kinase PpkA	99	99	98
wspD; hypothetical protein	100	100	100
wspA; chemotaxis transducer	98	99	99
wspE; chemotaxis sensor/effector fusion protein	99	99	100
probable methylesterase, WspF	100	100	99
wspR; two-component response regulator	99	100	100
sadC, protein SadC	99	100	100
siaD, protein SiaD	99	99	99
diguanylate cyclase,TpbB	99	99	99
roeA, Protein RoeA	98	100	98
mucR; signaling protein	99	99	100
bifA; protein BifA	100	100	100
transcriptional regulator, FleQ	100	100	100
pslB; biofilm formation protein PslB	99	99	99
pslC; biofilm formation protein PslC	99	99	99
pslD; biofilm formation protein PslD	100	100	100
pslE; biofilm formation protein PslE	99	99	99
pslF; biofilm formation protein PslF	99	99	99
pslG; biofilm formation protein PslG	100	100	100
pslH; biofilm formation protein PslH	99	99	99
pslI; biofilm formation protein PslI	100	99	99
pslJ; biofilm formation protein PslJ	100	100	100
pslK; biofilm formation protein PslL	98	99	99
pslL; hypothetical protein	99	100	99
pslM; biofilm formation protein PslM	99	99	99
pslN; biofilm formation protein PslN	99	100	99
pelA; hypothetical protein	99	93	99
pelB; pellicle/biofilm biosynthesis protein PelB	98	87	99
pelC; pellicle/biofilm biosynthesis protein PelC	100	95	100
pelD; pellicle/biofilm biosynthesis protein PelD	98	90	98
pelE; pellicle/biofilm biosynthesis protein PelE	97	90	99

**Name of the Genes/ Gene Products**	**Identity DMC-27b**	**Identity DMC-30b**	**Identity DMC-20c**
pelG; pellicle/biofilm biosynthesis transporter PelG	100	96	100
alginate biosynthesis protein, Alg44	100	100	100
trpE; anthranilate synthase component I	99	99	99
anthranilate synthetase component II, trpG	98	98	98
flgM; protein FlgM	100	99	100
fliA; flagellar biosynthesis sigma factor FliA	100	100	100
lasI; acyl-homoserine-lactone synthase	100	100	100
lasR; transcriptional regulator LasR	98	100	100
pppA; serine/threonine phosphatase PppA	99	99	99
stp1; phosphatase Stp1	100	100	100
clpV1; secretion protein ClpV1	99	99	99
hcpA; secreted protein Hcp	100	100	100
hcp1; protein secretion apparatus assembly protein	100	100	100
icmF1; type VI secretion protein IcmF	99	100	99
phnA; anthranilate synthase component I	98	98	98
pslA; biofilm formation protein PslA	100	99	100
algA; isomerase	100	99	100
fha1; Fha domain-containing protein	99	98	99
ladS; lost adherence sensor LadS	99	98	99

Table-5 Comparison of Biofilm related gene products of 3 *Pseudomonas aeruginosa* strains (in comparison with reference strain *Pseudomonas aeruginosa* PA01)

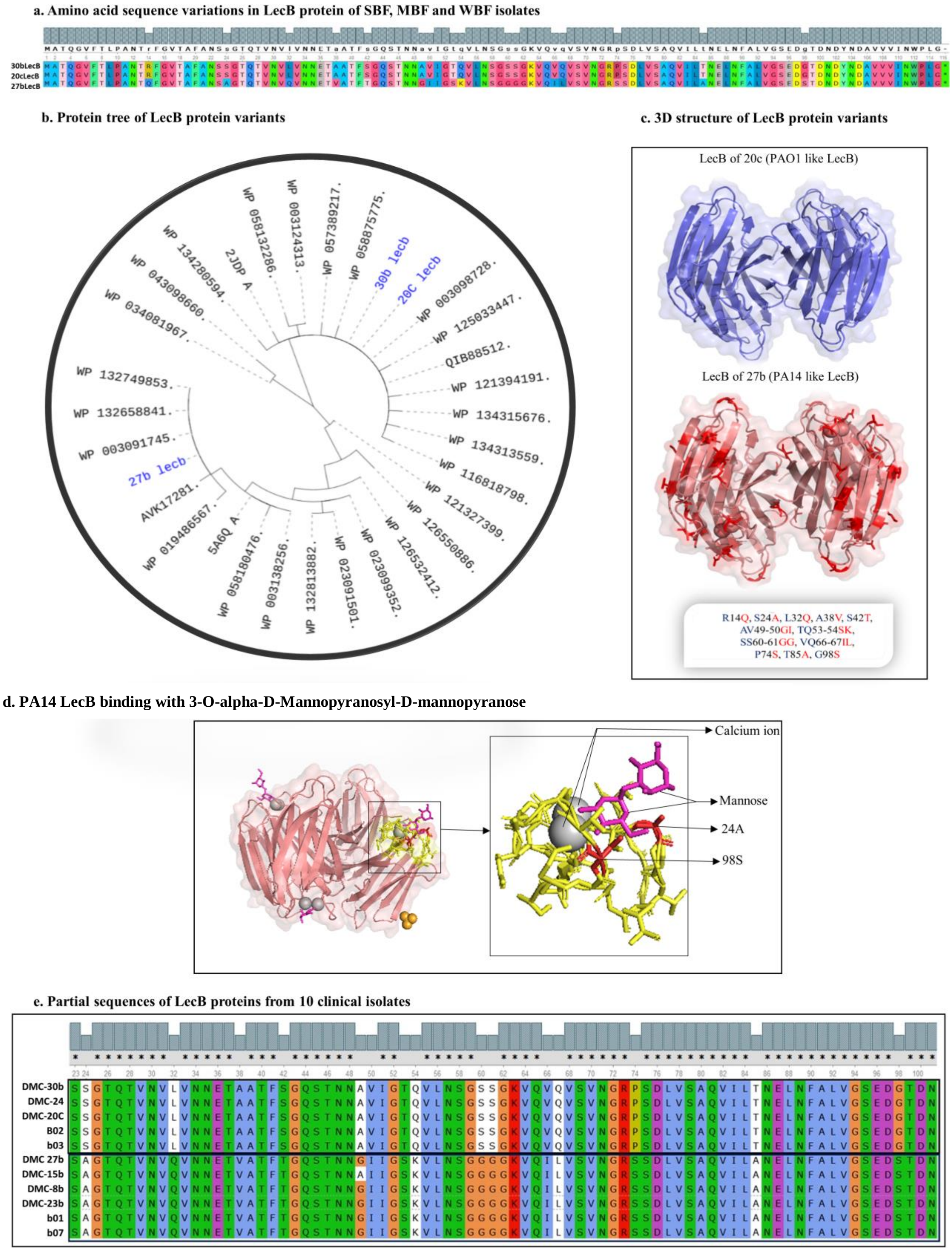

Supplementary Figure 1

In silico analysis of LecB protein from 30b (weak), 20c (moderate), 27b (strong) biofilm formers. (a) Protein sequence alignment of LecB from three isolates. (b) Phylogenetic analysis of

LecB protein in circular view. LecB proteins of our sequenced isolates marked with blue. Other LecB proteins were designated with their NCBI accession numbers. (c) 3D structure of the LecB protein visualized by PyMol. Upper structure shows LecB of PAO1 (slate blue), Lower structure shows LecB of 27b (salmon red) with mutation sites (red). (d) PA14 LecB binding with 3-O-alpha-D-Mannopyranosyl-D-mannopyranose. (e) Partial sequence of LecB (23-101) protein from 10 clinical isolates

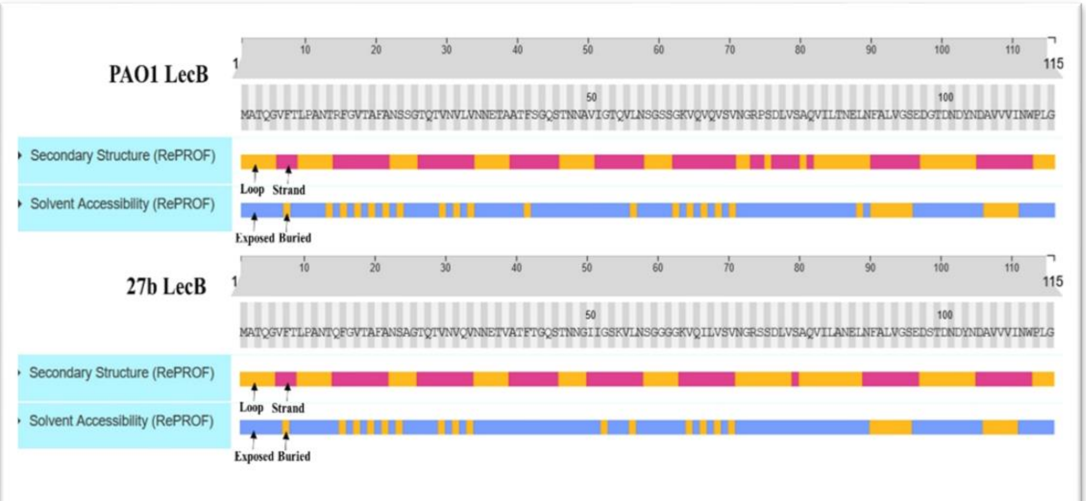

LecB	Helix	Strand	Loop	Buried	Exposed
Reference PA01	0%	62.61%	38.26%	24.35%	75.65%
27b	0%	61.74%	37.39%	23.48%	76.52%

Supplementary Figure 2: Secondary structure and Solvent Accessibility analysis of 27b LecB protein and Reference PAO1 LecB protein.

30b *pel*A

Description	Query Cover	E value	Per. Ident	Accession
Pseudomonas aeruginosa strain FDAARGOS_570 chromosome, complete...	100%	0.0	100.00%	CP033835.1
Pseudomonas aeruginosa PA7, complete genome	100%	0.0	99.82%	CP000744.1
Pseudomonas aeruginosa strain CR1 chromosome, complete genome	100%	0.0	99.36%	CP020560.1
Pseudomonas aeruginosa strain AR_0356 chromosome, complete genome	100%	0.0	99.36%	CP027169.1
Pseudomonas aeruginosa strain AR441 chromosome, complete genome	100%	0.0	99.33%	CP029093.1
Pseudomonas aeruginosa strain AZPAE15042 chromosome, complete gen...	100%	0.0	99.26%	CP041354.1
Pseudomonas aeruginosa strain PA-3 chromosome, complete genome	96%	0.0	92.83%	CP033084.1
Pseudomonas aeruginosa strain LW chromosome, complete genome	96%	0.0	92.79%	CP022478.1
Pseudomonas aeruginosa PA96 genome	96%	0.0	92.79%	CP007224.1
Pseudomonas aeruginosa strain FDAARGOS_505 chromosome, complete...	95%	0.0	92.93%	CP033832.1

Organism	Accession	*pel*A identity	*pel*B identity	*pel*C identity	*pel*D identity	*pel*E identity	*pel*F identity	*pel*G identity
***Pseudomonas aeruginosa* strain FDAARGOS_570**	CP033835.1	100	100	100	100	100	100	100
***Pseudomonas aeruginosa* PA7**	CP000744.1	99.82	99.78	99.81	99.85	99.8	99.93	99.85
***Pseudomonas aeruginosa* strain CR1**	CP020560.1	99.36	99.58	99.61	98.46	99.7	98.56	99.49
***Pseudomonas aeruginosa* strain AR_0356**	CP027169.1	99.36	99.53	99.61	98.61	99.6	98.56	99.2
***Pseudomonas aeruginosa* strain AR441**	CP029093.1	99.33	99.53	99.61	98.61	99.6	98.49	99.2
***Pseudomonas aeruginosa* strain AZPAE15042**	CP041354.1	99.26	99.64	99.81	98.54	99.6	98.69	99.49

Supplementary Table 6: NCBI nucleotide blast result for 30b *pel*A. The blue color box shows the strains that have 99-100% sequence homology with 30b *pel*A. Seven Pel operon genes of six *Pseudomonas aeruginosa* strains were compared with Pel operon genes of isolate 30b. The percentages of sequence identities are shown in the table.

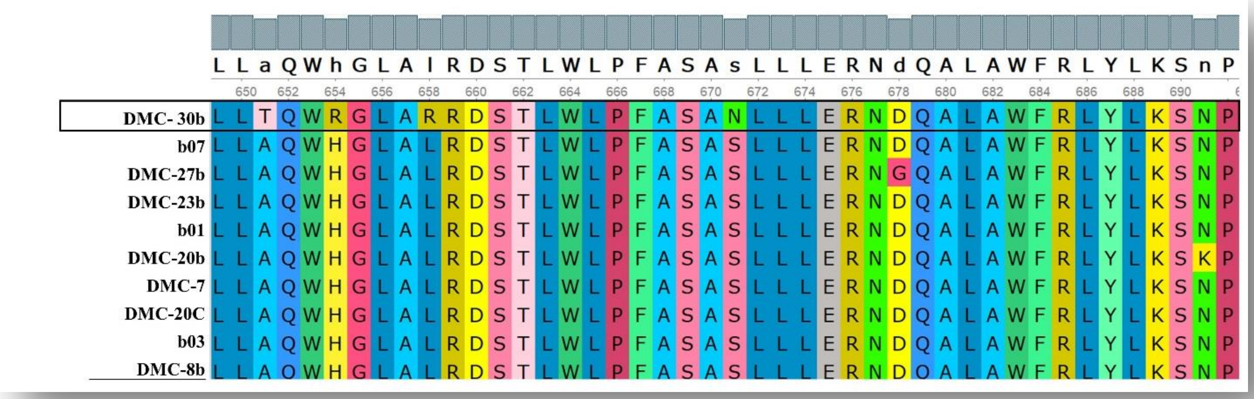

Supplementary Figure 3: Partial sequence of PelB (649-692) protein from 10 clinical isolates.

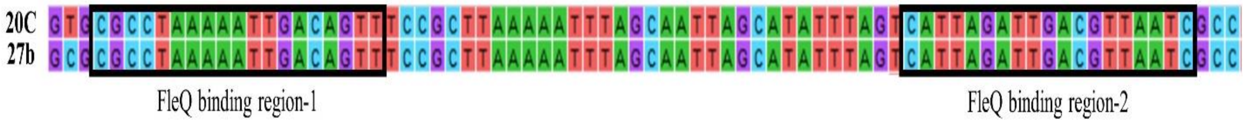

Supplementary Figure 4: Two FleQ binding sites in the promoter region of 27b *Pel*B and 20c *Pel*B.

Sample	***Target***	***ΔΔCt***	***RQ value***
27b_24hr	*pel*B	-2.355	5.116
30b_24hr	*pel*B	0	1
27b_24hr	*lec*B	-2.483	5.59
30b_24hr	*lec*B	0	1

Supplementary Table 6: RQ data table representing targets, ΔΔCt and RQ values
